# Knowledge and practices of Brazilian pediatric intensivists of PALICC-2 definitions and management of pediatric acute respiratory distress syndrome

**DOI:** 10.62675/2965-2774.20260056

**Published:** 2026-07-24

**Authors:** Ivone Maria da Rocha Meneguella, José Colleti Junior

**Affiliations:** 1 Faculdade de Medicina de Jundiaí Department of Pediatrics Jundiaí SP Brazil Department of Pediatrics, Faculdade de Medicina de Jundiaí - Jundiaí (SP), Brazil.

**Keywords:** Pediatric intensive care, Acute respiratory distress syndrome, Respiration, artificial, Guideline adherence, Surveys and questionnaires

## Abstract

**Objective:**

To evaluate the knowledge and clinical practices of pediatric intensivists in Brazil regarding the Pediatric Acute Lung Injury Consensus Conference 2 (PALICC-2) definitions and management of pediatric acute respiratory distress syndrome.

**Methods:**

A nationwide online survey was conducted between February and August 2025 among pediatric intensive care unit physicians. The questionnaire assessed demographics, training, knowledge of the PALICC-2 diagnostic criteria, and preferred management strategies in response to a case vignette. Responses were analyzed descriptively.

**Results:**

A total of 212 intensivists participated, of whom 158 completed the survey. Most respondents were female (67%), aged 31 - 40 years (50%), and had been out of school for 11 - 20 years (39%). The majority completed both general pediatrics and pediatric intensive care residency training (70%). For ventilatory management, 48.5% used a tidal volume of 4 - 6mL/kg, while 46.3% used 6 - 8mL/kg; 81% selected titration of optimal positive end-expiratory pressure; only 39% used a protective plateau pressure; 71.3% used a driving pressure limited to 15cmH_2_O. Analgesia strategies most frequently included neuromuscular blockade optimization (85%). In a case vignette, 88.8% correctly recognized severe pediatric acute respiratory distress syndrome according to PALICC-2 criteria, and nearly all (98.9%) classified the case as severe. In the event of deterioration despite optimized conventional therapies, 87% indicated extracorporeal membrane oxygenation as their rescue strategy.

**Conclusion:**

Brazilian pediatric intensivists demonstrated fair knowledge of PALICC-2 definitions, though they had some concerns about recognizing pediatric acute respiratory distress syndrome. Variability in management strategies underscores the need for continued dissemination of standardized guidelines and training to improve adherence and optimize outcomes.

## INTRODUCTION

Pediatric acute respiratory distress syndrome (PARDS) is a clinical syndrome of non-cardiogenic pulmonary edema and hypoxia that contributes to significant morbidity and mortality in pediatric intensive care units (ICUs).^(1,2)^ Children have historically been classified as having acute lung injury and acute respiratory distress syndrome (ARDS) based on the adult definitions originating from the 1994 American-European Consensus Conference (AECC), and 18 years later, the Berlin definitions, which were conducted without specific consideration of children.^(3,4)^ Both the AECC and Berlin ARDS definitions were focused on adult lung injury and have limitations when applied to children.^(3,4)^ These concerns prompted the organization of the Pediatric Acute Lung Injury Consensus Conference (PALICC) in 2015 and its recent update (PALICC-2), which established standardized definitions and management recommendations to improve diagnosis, comparability across studies, and patient outcomes.^(1,5)^

Despite these efforts, the extent to which intensivists worldwide – and specifically in Brazil – are familiar with and apply PALICC-2 recommendations remains uncertain. Recent studies have highlighted that implementation of the 2015 PALICC recommendations varies among pediatric ICUs,^(6,7)^ and nonadherence with recommendations is associated with higher mortality.^(8,9)^ Understanding knowledge gaps and practice variability is essential for designing educational strategies and fostering guideline adherence.

This study aimed to evaluate the knowledge and clinical practices of pediatric intensivists in Brazil regarding the PALICC-2 definitions and management of PARDS.

## METHODS

We conducted a cross-sectional, web-based survey between February and August 2025. Eligible participants were pediatric intensivists working in Brazilian pediatric ICUs. Invitations were distributed via the Brazilian Research Network in Pediatric Intensive Care (BRnet-PIC), which endorsed the study.^(10)^ Participation was voluntary and anonymous. Participants had to sign the consent form to respond to the survey, which comprised 10 demographic questions, 14 questions on knowledge of PARDS, 15 questions on the management of PARDS (treatment and clinical practices), and 1 vignette. The study was approved by the institutional research board of *Faculdade de Medicina de Jundiaí* (CAAE: 83001724.7.0000.5412), and all participants provided informed consent electronically.

### Survey instrument

The study used a structured questionnaire for data collection. The survey instrument was created by the authors in Brazilian Portuguese, inspired by the PALICC-2 publication. The instrument was tested according to the methodology of Burns et al.,^(11)^ and five experts from the BRnet-PIC pilot tested the survey and gave feedback on content and structure. The average completion time was 20 minutes. Their suggestions were analyzed and incorporated into the final version.

The questionnaire was distributed via electronic forms in REDCap, hosted at the *Faculdade de Medicina de Jundiaí*, to ensure data confidentiality and participant protection. It was distributed via WhatsApp by BRnet-PIC in February 2025, and a reminder was sent monthly until July 2025. The electronic case report forms (eCRFs) included: demographics (sex, age, years since graduation); medical training and specialization background; knowledge of PALICC-2 definitions; preferred ventilatory and pharmacological management strategies; rescue therapies for refractory PARDS, and a vignette to evaluate PARDS severity and management.

### Statistical analysis

Statistical analysis was conducted using STATA 16 (Stata Corp LP, TX, United States). Descriptive data were expressed as frequencies (%) for categorical variables and as medians and interquartile ranges (IQRs) for non-normal continuous variables. Spearman's rank correlation was used to assess the association between age and years of professional experience and the use of protective ventilation according to PALICC-2 recommendations. Statistical analysis was conducted using STATA 16 (Stata Corp LP, TX, United States).

## RESULTS

### Participants

Of the invited physicians (356), 212 responded to the survey; however, 54 did not complete most of the forms and were excluded, yielding a response rate of 44%. The majority of respondents were female (106/158; 67.1%) and between 31 and 40 years old (66/158; 41.8%). Most had 11 - 20 years since graduation (55/158; 34.8%). The majority (102/158; 64.6%) completed both general pediatrics and pediatric intensive care residency training, worked in São Paulo State (87/158; 55.1%), and in a pediatric ICU with exclusive pediatric patient admission (95/158; 60.1%). The demographic characteristics of participants are shown in table 1.

**Table 1:** Demographic and professional characteristics of participants

Variable		n (%)
Sex		106 (67.1)
	Female	
Age group (years)		
	< 30	9 (5.7)
	31 - 40	66 (41.8)
	41 - 50	40 (25.3)
	51 - 60	30 (19.0)
	> 60	7 (4.4)
Years since graduation		
	< 5	10 (6.3)
	5 - 10	33 (20.9)
	11 - 20	55 (34.8)
	21 - 30	27 (17.1)
	> 30	27 (17.1)
Professional background		
	No residency	7 (4.4)
	Pediatric residency	15 (9.5)
	Course in pediatric critical care	26 (16.5)
	Pediatric critical care residency	102 (64.6)
Type of pediatric ICU		
	Only pediatric	95 (60.1)
	Mixed (pediatric and adult)	3 (1.9)
	Mixed (pediatric and neonatal)	48 (30.4)
	Only neonatal	3 (1.9)
	Only pediatric cardiac	3 (1.9)
Number of pediatric ICU beds		
	< 10	51 (32.3)
	10 - 15	63 (39.9)
	16 - 20	28 (17.7)
	21 - 25	4 (2.5)
	≥ 26	6 (3.8)
Type of hospital		
	Pediatric/public/philanthropic	41 (25.9)
	Pediatric/private	12 (7.6)
	General/public/philanthropic	45 (28.5)
	General/private	43 (27.2)
	Other	10 (6.3)
State of Brazil		
	São Paulo	87 (57.2)
	Ceará	8 (5.3)
	Minas Gerais	7 (4.6)
	Pará	7 (4.6)
	Bahia	6 (3.9)
	Rio de Janeiro	6 (3.9)
	Paraná	5 (3.3)
	Santa Catarina	5 (3.3)
	Goiás	4 (2.6)
	Other	17 (11.3)

ICU - intensive care unit.

### Knowledge of PALICC-2 definitions

Only 31.6% (43/136) of respondents reported good knowledge of the PALICC-2 publication, followed by 28.7% who reported fair knowledge. Most physicians use chest X-rays to diagnose PARDS. For 29.4%, a bilateral infiltrate was required; for 49.3%, an infiltrate of one hemithorax was sufficient for the diagnosis of PARDS. Most (71/136; 52.2%) considered a period of ≥ 7 days for the new infiltrate in the diagnosis. For intubated patients on mechanical ventilation (MV), the majority reported using the oxygenation index for PARDS always (96/136; 70.6%) or sometimes (31/136; 22.8%).

We asked if it was possible to diagnose PARDS in patients on non-invasive ventilation (NIV); 90.4% agreed. We asked which MV mode they preferred for PARDS; 35.3% responded that there was no superior MV mode for PARDS, 30.1% preferred pressure control, and 22.8% preferred pressure-regulated volume control. Additionally, 48.5% used 4 - 6mL/kg and 46.3% used 6 - 8mL/kg of tidal volume, and 39% (53) used plateau pressure < 28cmH_2_O (< 32cmH_2_O if low thoracic compliance), and limit the driving pressure to 15cmH_2_O (97; 71.3%). We also asked if they used positive end-expiratory pressure (PEEP) titration during PARDS; 77.0% agreed, and they used the PEEP table (ARDSnet) sometimes (43/136, 31.6%) or always (38/136; 27.9%). We also asked which was the oxygen saturation target for a severe PARDS patient; 79.4% chose 88% to 92%, and 66.9% do permissive hypercapnia, tolerating a pH > 7.2. Table 1S (Supplementary Material) presents physicians’ responses on the diagnosis and management of patients with PARDS.

Table 2 shows the correlation of age range and working experience (years of graduation) with the answers for chest X-ray criteria for PARDS, and protective ventilation strategies. There was no statistical correlation.

**Table 2:** Spearman's correlation of age range and years of graduation with diagnosis criteria and protective ventilation

		Age range	Years of graduation	X-ray for diagnosis	Plateau pressure	Driving pressure
Age range						
	Spearman's rho	—				
	df*	—				
	p value	—				
Years of graduation						
	Spearman's rho	-0.466‡	—			
	df*	149	—			
	p value	< 0.001	—			
X-ray for diagnosis						
	Spearman's rho	0.087	-0.151	—		
	df*	131	131	—		
	p value	0.320	0.083	—		
Plateau pressure						
	Spearman's rho	0.009	-0.015	-0.238†	—	
	df*	150	150	134	—	
	p-value	0.911	0.854	0.005	—	
Driving pressure						
	Spearman's rho	0.085	0.010	-0.427‡	0.466‡	—
	df*	150	150	134	161	—
	p value	0.296	0.907	< 0.001	< 0.001	—

* df: degrees of liberty;

† p < 0.01;

‡ p < 0.001

### Management strategies

Nitric oxide is sometimes considered for PARDS patients, following an individualized evaluation, by 75.0% of respondents. Corticosteroids were considered effective after an individualized evaluation by 50.0% of respondents, although 33.0% reported not using them. Most respondents (84/132; 63.6%) reported that surfactant is not effective for PARDS. High-frequency oscillatory ventilation (HFOV) was considered effective by 59.1% of respondents following an individualized evaluation. Most participants (75/132; 56.8%) reported that the prone position is well tolerated by PARDS patients. Table 3 summarizes the responses.

**Table 3:** Opinions of Brazilian pediatric intensivists on treatments for severe pediatric acute respiratory distress syndrome

Intervention	Works very well n (%)	Works moderately (n %)	Depends on individual evaluation n (%)	Does not work n (%)
Inhaled nitric oxide	12 (9.1)	7 (5.3)	99 (75.0)	14 (10.6)
Corticosteroids	13 (9.8)	9 (6.8)	66 (50.0)	44 (33.3)
Surfactant	6 (4.5)	5 (3.8)	37 (28.0)	84 (63.6)
HFOV	26 (19.7)	20 (15.2)	78 (59.1)	8 (6.1)
Prone position	75 (56.8)	24 (18.2)	32 (24.2)	1 (0.8)

HFOV - high flow oscillatory ventilation.

### Clinical practices

Neuromuscular blockers were used sometimes (37/130; 28.5%) or always (62/130; 47.7%). However, train-of-four monitoring was unfamiliar to 43.8% of respondents and was not used by 36.2%. Regarding nutrition, 93.8% reported initiating early enteral feeding whenever feasible. For transfusion thresholds in patients with PARDS, most respondents (85/130; 65.4%) transfused when hemoglobin (Hb) < 7g/dL. To monitor lung volumes (e.g., tidal volume), most physicians reported using predicted body weight to calculate tidal volume sometimes (38/130; 29.2%) or always (71/130; 54.6%), remembering that PALICC-2 remarks that "the lesser of predicted body weight or actual body weight should be used". Capnography was used sometimes (21/130; 16.2%) or always (34/130; 26.2%).

Regarding the use of spontaneous breathing tests, most participants used them often (39/130; 30.0%) or always (65/130; 50.0%). Most of them use invasive arterial pressure monitoring in severe PARDS patients often (50/130; 38.5%) or always (26/130; 20.0%), and 78.5% observe the fluid accumulation during pediatric ICU stay.

Table 4 summarizes respondents’ clinical practices.

**Table 4:** Clinical practice survey results

Clinical practice		n (%)
Use of neuromuscular blockade with sedoanalgesia to achieve lung-protective ventilation targets		
	Never	4 (3.1)
	Rarely	6 (4.6)
	Sometimes	37 (28.5)
	Often	62 (47.7)
	Always	21 (16.2)
Use of train-of-four to guide neuromuscular blockade level		
	Do not know what train-of-four is	57 (43.8)
	Do not use	47 (36.2)
	Sometimes	19 (14.6)
	Often	7 (5.4)
	Always	0 (0.0)
Nutritional management		
	Keep NPO until stabilized, regardless of time	1 (0.8)
	Initiate early enteral nutrition whenever possible	122 (93.8)
	Keep NPO longer and initiate parenteral nutrition after 5 days	4 (3.1)
	Initiate with low protein supply (< g/kg/day)	3 (2.3)
Hemoglobin threshold for transfusion in hemodynamically stable patients		
	Transfuse if Hb < 9g/dL	10 (7.7)
	Transfuse if Hb < 8g/dL	23 (17.7)
	Transfuse if Hb < 7g/dL	85 (65.4)
	Transfuse if Hb < 6g/dL	7 (5.4)
	Transfuse if Hb < 5g/dL	5 (3.8)
Use of predicted body weight for tidal volume calculation		
	Do not know how to calculate it	8 (6.2)
	Do not use	13 (10.0)
	Sometimes use	38 (29.2)
	Always use	71 (54.6)
Use of capnography		
	Never	16 (12.3)
	Rarely	13 (10.0)
	Sometimes	21 (16.2)
	Often	46 (35.4)
	Always	34 (26.2)
Performance of extubation readiness and spontaneous breathing trials		
	Never	4 (3.1)
	Rarely	5 (3.8)
	Sometimes	17 (13.1)
	Often	39 (30.0)
	Always	65 (50.0)
Use of invasive arterial pressure monitoring		
	Never	11 (8.5)
	Rarely	14 (10.8)
	Sometimes	29 (22.3)
	Often	50 (38.5)
	Always	26 (20.0)
Monitoring of cumulative fluid balance		
	Never	1 (0.8)
	Rarely	4 (3.1)
	Sometimes	2 (1.5)
	Often	21 (16.2)
	Always	102 (78.5)

NPO - Nil Per Os; Hb - hemoglobin.

### Case scenarios

**Figure 1:**
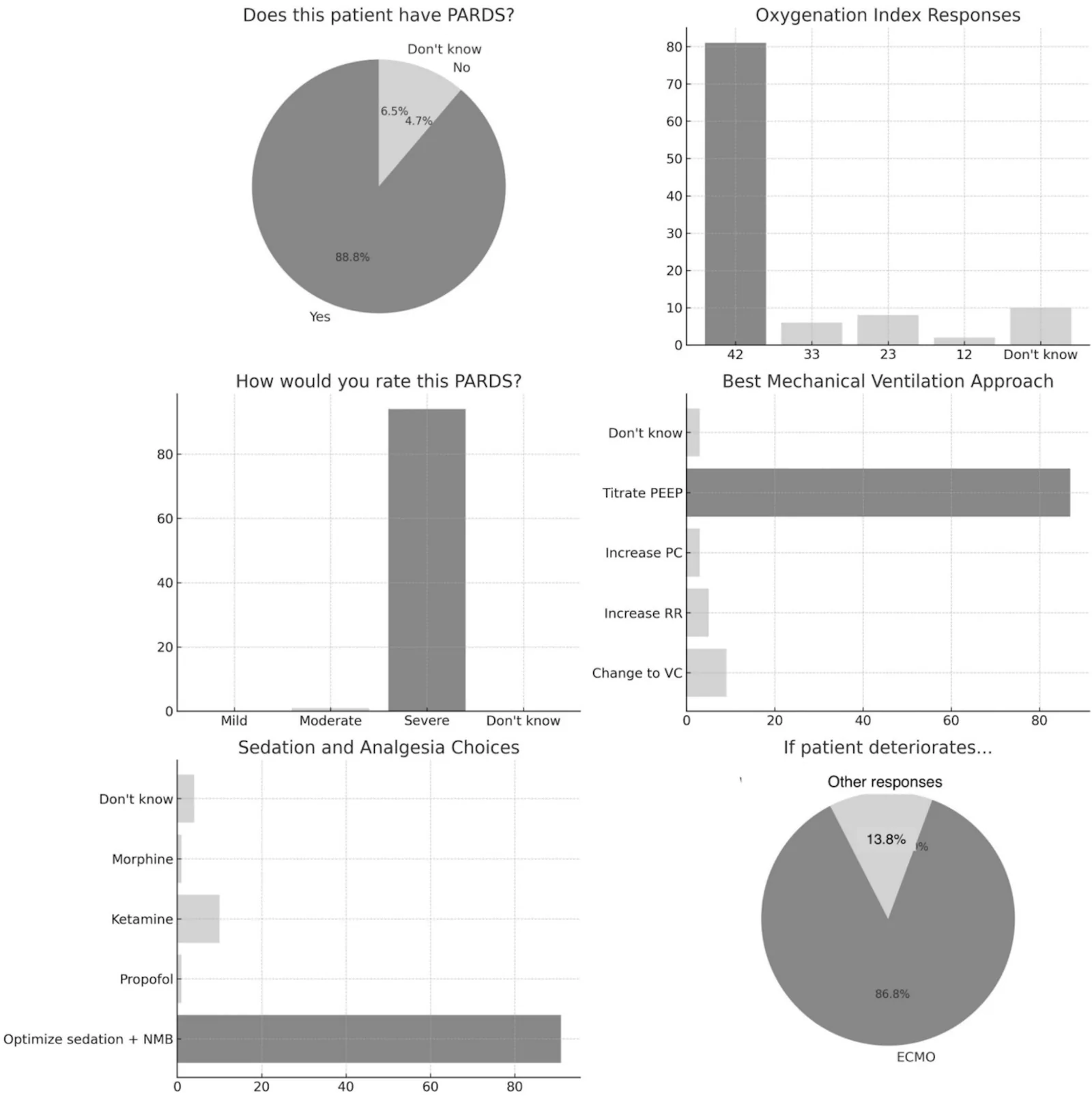
Correct response s regarding the diagnosis and management of a hypothetical patient.

The case scenario is depicted in figure 1, reporting a hypothetical patient with severe PARDS and questions regarding the patient's diagnosis and management. The correct rate of responses is embedded in the figure. In the vignette, a severe PARDS case, 88.8% of respondents chose the correct option, 75.7% correctly predicted the oxygenation index, and 86.8% would recommend extracorporeal membrane oxygenation (ECMO) if the patient continued to deteriorate clinically despite optimization of all medical procedures.

Case presentation and questions: A 6-year-old female patient, diagnosed with pneumococcal pneumonia, intubated on mechanical ventilation (fraction of inspired oxygen of 1.0, positive endexpiratory pressure of 5, pressure controlled of +25, respiratory rate of 30), pressurecontrolled mode. Sedated with dexmedetomidine (1.0) and fentanyl (2) with Richmond Agitation-Sedation Scale +1. Arterial blood gas: pH: 7.08, partial pressure of carbon dioxide of: 120, partial pressure of oxygen of 60, bicarbonate: 25, blood oxygen saturation 82%, mean airway pressure: 25cmH2O. Chest X-ray showed consolidation throughout the right hemithorax and diffuse infiltrate in the left hemithorax. Chest ultrasound showed laminar effusion with an area of consolidation on the right and B lines on the left. Do you think this patient has pediatric acute respiratory distress syndrome? How would you rate the severity of this pediatric acute respiratory distress syndrome? What is the oxygenation index? What is the best course of action regarding mechanical ventilation? What is the best approach related to sedation and analgesia? If the patient continues to deteriorate, optimizing sedation/analgesia, mechanical ventilation parameters, fluid balance, prone positioning, and positive end-expiratory pressure titration guided by electrical impedance tomography, what would be your course of action?

PARDS - pediatric acute respiratory distress syndrome; PEEP - positive end-expiratory pressure; PC - pressure controlled; RR - respiratory rate; VC - volume controlled; NMB - neuromuscular blockade; ECMO - extracorporeal membrane oxygenation.

## DISCUSSION

In this nationwide survey of Brazilian pediatric intensivists, we found significant variability in knowledge and application of the PALICC-2 recommendations for the diagnosis and management of PARDS. Although most respondents reported familiarity with PALICC-2, important gaps and inconsistencies were evident, particularly regarding diagnostic criteria and ventilatory management strategies. These findings highlight the challenges of translating updated international guidelines into everyday clinical practice.

One of the most striking results was the frequent reliance on chest radiography for PARDS diagnosis, with a considerable proportion of intensivists requiring bilateral infiltrates. This contrasts with PALICC-2, which allows for diagnosis with unilateral infiltrates and even supports a category of "possible PARDS" to account for children managed with noninvasive modalities such as NIV or high-flow nasal cannula. Furthermore, almost half of the respondents did not consider a 7-day window acceptable for new infiltrates, whereas the consensus definition recommends it within 7 days of a known clinical insult. This concept stems from the 2015 PALICC, which highlighted a knowledge gap in the diagnostic criteria for PARDS.

Regarding oxygenation metrics, most participants reported routine or occasional use of the oxygenation index, in line with PALICC-2, which endorses the oxygenation index and oxygenation saturation index as more reliable severity markers than partial pressure of oxygen in arterial blood against the fraction of inspired oxygen (PaO_2_/FiO_2_) ratios in mechanically ventilated children. Interestingly, only 34.6% abandoned PaO_2_/FiO_2_ and migrated to oxygenation index and oxygenation saturation index. Encouragingly, the vast majority agreed that PARDS can be diagnosed in patients on noninvasive ventilation, as already reported in PALICC (2015), but without severity stratification.

Ventilatory management also showed heterogeneity. Most respondents reported using lung-protective ventilation (LPV) with tidal volumes of 6 - 8mL/kg (and 4 - 6mL/kg when needed to keep plateau and driving pressures within suggested limits), broadly consistent with PALICC-2 guidance. However, only 39% reported routinely limiting plateau and driving pressures, indicating a gap between recommended pressure targets and reported bedside practice, and identifying a potential priority area for focused education and implementation efforts. Recruitment maneuvers and PEEP titration strategies were also variably applied, with frequent use of ARDSNet tables, in line with PALICC-2 recommendations. Notable gaps were identified in the monitoring of neuromuscular blockade (with limited use of train-of-four) and in the routine application of capnography, suggesting opportunities to optimize adherence to safety standards. Neuromuscular blockade in PARDS primarily aims to control excessive respiratory effort and mitigate patient self-inflicted lung injury (P-SILI), whilst permissive hypercapnia is a ventilatory strategy directly linked to limiting plateau pressure and driving pressure.^(9)^

Use of adjunctive therapies was also inconsistent. Prone positioning was widely perceived as beneficial, although PALICC-2 "cannot recommend for or against prone positioning". At the same time, nitric oxide, corticosteroids, and surfactant were mostly considered only after individualized assessment, reflecting both the uncertainty of supporting evidence and the cautious approach in clinical practice. High-frequency oscillatory ventilation was reported as a selective option depending on individual evaluation, which aligns with the guidelines that "cannot make a recommendation as to whether HFOV should be used instead of conventional ventilation in patients with PARDS.

Supportive care practices were more consistent with international recommendations. The majority reported early initiation of enteral nutrition, restrictive transfusion thresholds, and monitoring of cumulative fluid balance, all of which are endorsed by PALICC-2.

A vignette was included in the survey to better assess respondents’ daily practices. The case scenario showed a patient with severe PARDS. Most respondents (88.8%) reported that the patient had PARDS, correctly classified it as severe PARDS (98.9%), chose the correct oxygenation index (75.7%), titrated the optimal PEEP (81.3%), optimized sedation (85%), and correctly indicated ECMO (86.8%).

Only a few studies address adherence to PALICC guidelines. Kopstick et al. found that provider recognition of PARDS was low (30% of cases) and that adherence to key PALICC-1 LPV recommendations was poor and did not correlate with provider recognition of the syndrome.^(12)^ In a 2023 study, patients with acute hypoxic respiratory failure who received invasive MV were retrospectively evaluated to determine whether the patient met the PALICC definition of moderate to severe PARDS and rated their diagnostic confidence.^(13)^ The interrater reliability for diagnosing moderate to severe PARDS was substantial, with diagnostic disagreements commonly caused by differences in chest radiograph interpretations, which can be challenging sometimes if detached from the whole clinical presentation.

Our findings should be interpreted in light of some limitations. First, participation was voluntary through an invitation from BRnet-PIC, which could introduce selection bias, as intensivists more familiar with PALICC-2 may have been overrepresented. Another possible selection bias was the fact that 57.2% of respondents were from São Paulo State, which may be overrepresented. However, it is known that this state comprises more pediatric ICUs than any other State in Brazil. These factors may prevent the generalization of our findings to all Brazilian settings. We had 54 physicians who started the questionnaire but did not continue, and we don't know the profile of these participants, but this may have also introduced some degree of selection bias into our study. The survey assessed self-reported practices and responses to vignettes, which may not fully reflect real-world decision-making. Also, the response rate was suboptimal (44%), which prevents the generalization of the results. The study was conducted in Brazil, and results may not represent other settings, particularly those with different resource constraints. Finally, the online survey nature precluded objective assessment of actual bedside adherence.

The identified knowledge gaps highlight the need for targeted dissemination strategies, continuing medical education, and institutional protocols to support the integration of PALICC-2 into clinical practice. In particular, clarifying diagnostic definitions, reinforcing LPV strategies, and standardizing the use of monitoring tools may improve adherence to evidence-based care. Future studies should evaluate whether structured educational interventions and protocolized approaches can improve consistency and patient outcomes.

## CONCLUSION

This nationwide survey demonstrates that Brazilian pediatric intensivists have moderate knowledge of the gaps in the updated PALICC-2 recommendations and exhibit heterogeneous application of these recommendations in daily practice. Key gaps include reliance on outdated diagnostic imaging criteria, inconsistent use of lung-protective ventilation, and variable adoption of monitoring strategies. Broader dissemination of PALICC-2, structured educational initiatives, and institutional protocols are needed to enhance adherence to evidence-based care. Strengthening alignment between guidelines and practice may ultimately improve outcomes for children with pediatric acute respiratory distress syndrome.

## Data Availability

The contents will be made available at the time of publication of the article.

## References

[1] Emeriaud G, López-Fernández YM, Iyer NP, Bembea MM, Agulnik A, Barbaro RP (2023). Second Pediatric Acute Lung Injury Consensus Conference (PALICC-2) Group on behalf of the Pediatric Acute Lung Injury and Sepsis Investigators (PALISI) Network. Executive Summary of the Second International Guidelines for the Diagnosis and Management of Pediatric Acute Respiratory Distress Syndrome (PALICC-2). Pediatr Crit Care Med.

[2] Quasney MW, López-Fernández YM, Santschi M, Watson RS, Pediatric Acute Lung Injury Consensus Conference Group (2015). The outcomes of children with pediatric acute respiratory distress syndrome: proceedings from the Pediatric Acute Lung Injury Consensus Conference. Pediatr Crit Care Med.

[3] Bernard GR, Artigas A, Brigham KL, Carlet J, Falke K, Hudson L (1994). The American-European Consensus Conference on ARDS. Definitions, mechanisms, relevant outcomes, and clinical trial coordination. Am J Respir Crit Care Med.

[4] Ranieri VM, Rubenfeld GD, Thompson BT, Ferguson ND, Caldwell E, Fan E (2012). ARDS Definition Task Force. Acute respiratory distress syndrome: the Berlin Definition. JAMA.

[5] Pediatric Acute Lung Injury Consensus Conference Group (2015). Pediatric acute respiratory distress syndrome: consensus recommendations from the Pediatric Acute Lung Injury Consensus Conference. Pediatr Crit Care Med.

[6] Khemani RG, Smith L, Lopez-Fernandez YM, Kwok J, Morzov R, Klein MJ (2019). Pediatric Acute Respiratory Distress syndrome Incidence and Epidemiology (PARDIE) Investigators; Pediatric Acute Lung Injury and Sepsis Investigators (PALISI) Network. Paediatric acute respiratory distress syndrome incidence and epidemiology (PARDIE): an international, observational study. Lancet Respir Med.

[7] Rowan CM, Klein MJ, Hsing DD, Dahmer MK, Spinella PC, Emeriaud G (2020). Early Use of Adjunctive Therapies for Pediatric Acute Respiratory Distress Syndrome: A PARDIE Study. Am J Respir Crit Care Med.

[8] Khemani RG, Parvathaneni K, Yehya N, Bhalla AK, Thomas NJ, Newth CJ (2018). Positive End-Expiratory Pressure Lower Than the ARDS Network Protocol Is Associated with Higher Pediatric Acute Respiratory Distress Syndrome Mortality. Am J Respir Crit Care Med.

[9] Bhalla AK, Klein MJ, Emeriaud G, Lopez-Fernandez YM, Napolitano N, Fernandez A (2021). Pediatric Acute Respiratory Distress Syndrome Incidence and Epidemiology (PARDIE) V.2. Investigators and Pediatric Acute Lung Injury and Sepsis Investigators (PALISI) Network. Adherence to lung-protective ventilation principles in pediatric acute respiratory distress syndrome: a pediatric acute respiratory distress syndrome incidence and epidemiology study. Crit Care Med.

[10] Brazilian Research Network in Pediatric Intensive Care (BRnetPIC) About us.

[11] Burns KE, Duffett M, Kho ME, Meade MO, Adhikari NK, Sinuff T (2008). ACCADEMY Group. A guide for the design and conduct of self-administered surveys of clinicians. CMAJ.

[12] Kopstick AJ, Rufener CR, Banerji AO, Hudkins MR, Kirby AL, Markwardt S (2022). Recognizing Pediatric ARDS: Provider Use of the PALICC Recommendations in a Tertiary Pediatric ICU. Respir Care.

[13] Silver L, Kaplan D, Asencio J, Mandell I, Fishbein J, Shah S (2023). Interrater Reliability of the 2015 Pediatric Acute Lung Injury Consensus Conference Criteria for Pediatric ARDS. Chest.

